# Supplementary material for: Novel Driver Strength Index highlights important cancer genes in TCGA PanCanAtlas patients
Source: PeerJ. 2022 Aug 11;10:e13860. doi: 10.7717/peerj.13860 (PMC9375969; doi:10.7717/peerj.13860)
Supplement: Supplemental Information 2 [file peerj-10-13860-s002.zip › COHORTS/genes plots/detailed/2021_8_16_14_9_distribution_events_detailed_44.pdf]

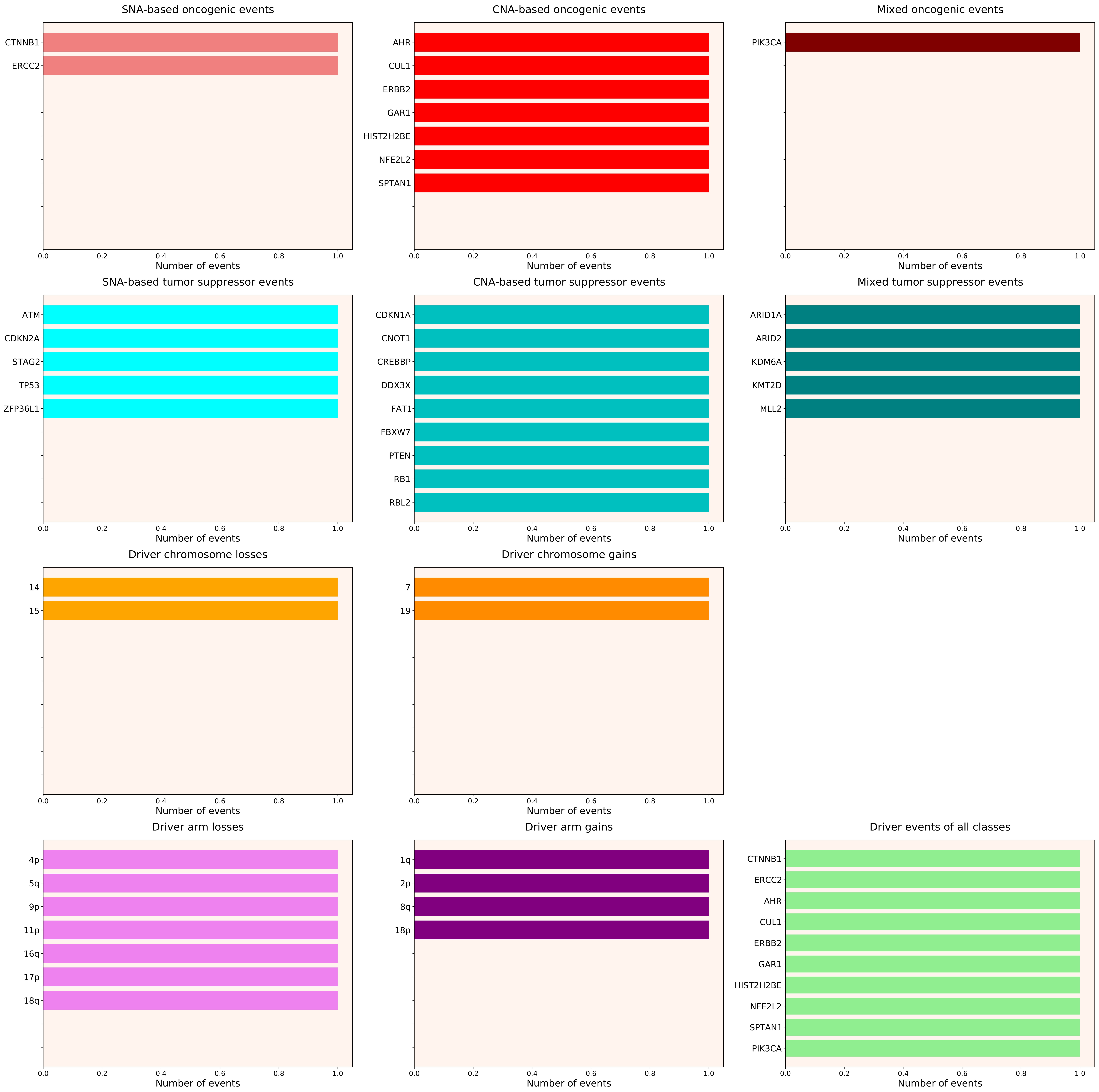
SNA-based oncogenic events
CTNNB1
ERCC2
0.0 0.2 0.4 0.6 0.8 1.0
Number of events
CNA-based oncogenic events
AHR
CUL1
ERBB2
GAR1
HIST2H2BE
NFE2L2
SPTAN1
0.0 0.2 0.4 0.6 0.8 1.0
Number of events
Mixed oncogenic events
PIK3CA
0.0 0.2 0.4 0.6 0.8 1.0
Number of events
SNA-based tumor suppressor events
ATM
CDKN2A
STAG2
TP53
ZFP36L1
0.0 0.2 0.4 0.6 0.8 1.0
Number of events
CNA-based tumor suppressor events
CDKN1A
CNOT1
CREBBP
DDX3X
FAT1
FBXW7
PTEN
RB1
RBL2
0.0 0.2 0.4 0.6 0.8 1.0
Number of events
Mixed tumor suppressor events
ARID1A
ARID2
KDM6A
KMT2D
MLL2
0.0 0.2 0.4 0.6 0.8 1.0
Number of events
Driver chromosome losses
14
15
0.0 0.2 0.4 0.6 0.8 1.0
Number of events
Driver chromosome gains
7
19
0.0 0.2 0.4 0.6 0.8 1.0
Number of events
Driver arm losses
4p
5q
9p
11p
16q
17p
18q
0.0 0.2 0.4 0.6 0.8 1.0
Number of events
Driver arm gains
1q
2p
8q
18p
0.0 0.2 0.4 0.6 0.8 1.0
Number of events
Driver events of all classes
CTNNB1
ERCC2
AHR
CUL1
ERBB2
GAR1
HIST2H2BE
NFE2L2
SPTAN1
PIK3CA
0.0 0.2 0.4 0.6 0.8 1.0
Number of events
